# Mechanical hyperinflation maneuver and intracranial compliance of critical neurological patients: protocol for a randomized controlled equivalence trial

**DOI:** 10.1186/s13063-023-07362-5

**Published:** 2023-05-22

**Authors:** Daniela de Almeida Souza, Marina Wolff Branco, Hipólito Carraro Junior, Ana Márcia Delattre Zocolotti, Sibele Yoko Mattozo Takeda, Silvia Valderramas

**Affiliations:** 1 Internal Medicine and Health Sciences, Universidade Federal Do Parana, Avenida Coronel Francisco H. Dos Santos, 100, Caixa Postal 19031, Centro Politécnico, Jardim das Américas, Curitiba, PR 81531-980 Brazil; 2 Physiotherapist from Empresa Brasileira de Serviços Hospitalares, Rio de Janeiro, Brazil; 3 Intensive Care Unit, Complexo Hospital de Clinicas, Curitiba, PR Brazil; 4 Department of Prevention and Rehabilitation in Physiotherapy of the Universidade Federal Do Parana, Curitiba, PR Brazil; 5 Internal Medicine and Health Sciences and Department of Prevention and Rehabilitation in Physical Therapy, Universidade Federal Do Parana, Curitiba, PR Brazil

**Keywords:** Mechanical ventilators, Stroke, Intracranial pressure, Respiratory mechanics, Physical therapy techniques, Randomized controlled trial, Intracranial compliance

## Abstract

**Background:**

Mechanical hyperinflation maneuver (MHM) is a technique known for optimizing bronchial hygiene and respiratory mechanics; however, its effects on intracranial compliance are not known.

**Methods:**

Sixty patients aged ≥ 18 years, with clinical diagnosis of acute stroke, confirmed by neuroimaging examination, with onset of symptoms within 72 h, under mechanical ventilation through tracheal tube, will participate in this study. Participants will be randomly allocated into 2 groups: experimental group (*n* = 30)—MHM plus tracheal aspiration—and control group (*n* = 30)—tracheal aspiration only. Intracranial compliance will be measured by a non-invasive technique using Brain4care BcMM-R-2000 sensor. This will be the primary outcome. Results will be recorded at 5 times: T0 (start of monitoring), T1 (moment before MHM), T2 (moment after the MHM and before tracheal aspiration), T3 (moment after tracheal aspiration), T4, and T5 (monitoring 10 and 20 min after T3). Secondary outcomes are respiratory mechanics and hemodynamic parameters.

**Discussion:**

This study will be the first clinical trial to examine the effects and safety of MHM on intracranial compliance measured by non-invasive monitoring. Limitation includes the impossibility of blinding the physical therapist who will supervise the interventions. It is expected with this study to demonstrate that MHM can improve respiratory mechanics and hemodynamic parameters and provide a safe intervention with no changes in intracranial compliance in stroke patients.

**Supplementary Information:**

The online version contains supplementary material available at 10.1186/s13063-023-07362-5.

## Introduction

Neurocritical patients with acute neurological impairment, especially stroke, often require the use of mechanical ventilation (MV) [1, 2], either by lowering of the level of consciousness (due to lesion in the thalamus, limbic system, reticular formation in the brainstem), protection of the airways (brainstem), or respiratory control (respiratory center in the cortex, bulb, and pons) [3].

Prolonged MV dependence associated with brain-lung crosstalk and immunosuppression induced by ischemic event [2, 4, 5] can increase the retention of lung secretions and lead to pulmonary complications with an underlying higher risk of death [2].

Evidence corroborates the benefits of using strategies for the removal of secretion associated with MV [6, 7], including MHM, which has been widely applied and discussed [8, 9]. However, hyperinflation causes higher intrathoracic pressure (ITP), which can influence cardiac output (CO), mean arterial pressure (MAP), and intracranial pressure (ICP) [10], thus compromising the indication of this technique. Even though some studies have demonstrated cardiovascular stability during MHM [11, 12], no studies have addressed the monitoring of ICP or intracranial compliance (ICC) during and after the maneuver performance in critical neurological patients. This possibly occurs due to the barrier imposed by the invasive method involving skull trepanning and insertion of a catheter in the cranium, required for monitoring the ICP in traditional methods [13]. Although the technique of inserting a cerebral catheter is the gold standard and most recognized measure for ICP monitoring, the exigence of surgical intervention, training, and specific interpretation technique make it financially unviable for studies at intensive care units (ICUs) on a small and large scale.

Over the past few years, a new instrument has been proposed for non-invasive monitoring of ICC through a sensor capable to detect bone cranial deformation derived from ICP variation [14, 15], which allowed to monitor these patients, called Brain4care (B4C) sensor. The method is in good agreement with the invasive method, the gold standard for the detection of intracranial hypertension [16].

The B4C technology allows analyzing the ICP waveform, composed of the components (peaks) P1, P2, and P3, and the relationship between these different components provides information about the ICC. ICC is characterized by the relationship between the volumes of intracranial components (brain, cerebrospinal fluid, and blood). The loss of hemostasis between these components is related to a substantial increase in ICP [15].

In this context, considering the need to perform MV in critical neurological patients, as well as the retention and need for effective, safe removal of lung secretions and related clinical impact on ICC variation, our objective was to develop an MHM protocol and investigate its effects on ICC and respiratory mechanics on patients with stroke.

## Methods

### Trial design

The protocol complies with a randomized controlled equivalence trial, double-blinded, parallel (1:1 ratio), and exploratory approved by the Research Ethics Committee of the Hospital de Clínicas of the Universidade Federal do Parana (CAAE: 01,378,118.7.0000.0096). The individuals are allocated through simple randomization into two groups. All items in the Data Set of the Register of Tests by the WHO were recorded on the database of the Brazilian Clinical Trial Registry (RBR – 5qs9k2n). Any alteration in the protocol is informed to the above-mentioned bodies. When preparing this protocol, we used the SPIRIT reporting guidelines [17], and a table with the standard protocol items (SPIRIT) is presented in Table 1. The SPIRIT checklist is available as an additional file (Additional file 1).

**Table 1:** Time schedule of enrollment, interventions, assessments, and visits for participants according to Recommendations for Interventional Trials (SPIRIT)

		**Study period**						
		**Enrollment**	**Allocation**	**Post-allocation**			**Close-out**	
**Time point**		**-t1**	**T0**	**T1**	**T2**	**T3**	**T4**	**T5**
**Enrollment**								
Eligibility screen		X						
Informed consent		X						
Allocation			X					
**Interventions**								
Control group					X	X		
Experimental group					X	X		
**Outcomes variables**								
**Background information**								
Socio-demographic and clinical characteristics		X						X
**Primary outcome measures**								
Intracranial compliance	Relation P2/P1			X	X	X	X	X
	TTP			X	X	X	X	X
	Pulse amplitude							
**Secondary outcome measures**								
Respiratory mechanics	Static compliance			X	X	X	X	X
	Dynamic compliance			X	X	X	X	X
	Resistance			X	X	X	X	X
	Expired current volume							
Hemodynamic stability	MAP			X	X	X	X	X
	HR			X	X	X	X	X
	SpO_2_			X	X	X	X	X

*TTP* time to peak, *MAP* mean arterial pressure, *HR* heart rate, *SpO*_*2*_ peripheral oxygen saturation

### Setting and participants

Information and guidelines on this research (objectives, methodology, risks, and benefits) are given to critical neurological patients admitted to ICU at the Clinical Hospital Complex of the Federal University of Paraná and met the inclusion criteria. The term of consent will be obtained by one of the researchers of the group, before the admission of the participant in the research. Their inclusion in the study is effective by agreeing to participate and signing an Informed Consent (IC), according to Resolution 466/2012 by the National Health Council This researcher will make the initial contact with the person responsible for the participant, explaining in detail all the procedures that will be performed.

Individuals with acute stroke diagnosis are included in the study upon neuroimage exam confirmation, with symptoms dating back to 72 h, under MV through a tracheal tube, both male and female and over the age of 18, whose relatives signed the IC. Exclusion criteria included individuals who received contraindication for the enhancement of positive ITP (pneumothorax, hemothorax, and acute respiratory distress syndrome), presented peak pressure (Ppeak) > 40 cmH_2_O in the MV, patients with Richmond Agitation-Sedation Scale (RASS) above -3 (asynchronicity in MV and difficulty in coupling to the ICP monitoring sensor), patients with hemodynamic instability according to higher concentrations of vasoactive amines over the last 12 h, and those who had been subjected decompressive craniectomy.

The following strategies will be carried out for achieving adequate participant: dissemination of the research among members of the multidisciplinary team, training of physiotherapists to screen potential patients for the study, and actively search for patients in intensive care units.

To control possible confusing factors regarding ICP increase, we will assess and monitor the level of carbon gas (CO_2_) exhaled – end-tidal CO_2_ (ETCO_2_) and pain throughout the data collection. ETCO_2_ assessment is performed using a capnograph (*Dräger Vamos@ plus* monitor), whose sensors are calibrated 2 h before the collection period and alarms, reference measures, and oxygen compensation adjusted [2]. Pain is assessed according to the Behavioral Pain Scale, and the evaluation tool focused on behavioral indicators of pain previously translated and adapted to Portuguese [18, 19]. All researchers were properly trained for data collection.

### Randomization and allocation concealment

The participants are randomly allocated to two groups using a simple randomization procedure with computerized random numbers (Randomizer software program—Version 4.0). Participant allocation was conducted using sequentially numbered, opaque and sealed envelopes, opened only at the time of participant’s inclusion in the study. Randomization and allocation procedures were conducted by an independent physical therapist not involved with assessment or interventions, same for recruitment.

### Data management

Prior to the beginning of the trial, study personnel will undergo training sessions on data collection and will be individually tested on data entry as well as outcome assessments. Study data will be collected on a paper form and transferred to an excel spreadsheet. To ensure the integrity of the data, regular quality checks will be performed to assess for missing data and invalid field entries.

All electronic study data will be stored on a secure terminal server at the Department of Prevention and Rehabilitation in Physical Therapy, Universidade Federal do Parana, being access limited only to authorized staff, encrypted and password-protected with limited access. All electronic information will be recorded using study identification numbers, rather than participant names.

All data and all source documents, as well as the consent forms, will be filed archived at in a cabinet with restricted access to members of the research group.

### Interventions

**Fig. 1:**
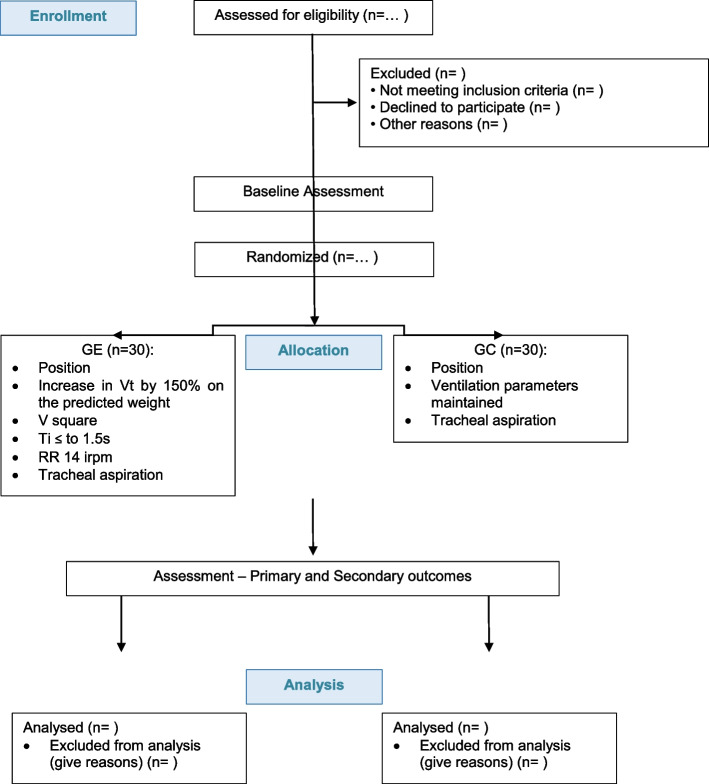
Flowchart of recruitment, interventions, and evaluations of participants. EG, experimental group; CG, control group; Vt, tidal volume; V, flow; RR, respiratory rate; Ti, inspiratory time

**Fig. 2:**
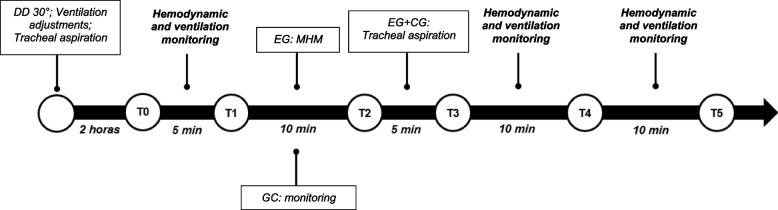
Schematic drawing of experimental design. T0 to T5 represents the assessment stages. DD, dorsal decubitus; EG, experimental group; MHM, mechanical hyperinflation maneuver; CG, control group

After the assessments, the selected patients are randomly allocated into two groups (Fig. 1): experimental group (EG)—composed of individuals who will be subjected to MHM + tracheal aspiration—and control group (CG)—only tracheal aspiration intervention. All individuals will be positioned on dorsal decubitus at the bedside at 30°; MV is adjusted on assisted ventilation mode, controlling volume, tidal volume (Vt) of 6 ml/Kg of predicted weight, positive expiratory-end pressure (PEEP) 8 cmH_2_O, inspired oxygen fraction (FIO_2)_, peripheral oxygen saturation (SpO_2_) of 94%, and respiratory rate (RR) and flow (V) adjusted to maintain ventilation synchrony and gasometric demands. We will use a number 14 closed aspiration system with verified cuff pressure and the occurrence of leaks. All participants will be subjected to tracheal aspiration, and only 2 h later, the data collection is initiated (Fig. 2). During these 2 h, patients will only be monitored and maintained on the initial position; no concomitant procedures or disconnection of MV circuit are conducted. In case it is not possible to maintain such conditions, the collection is suspended and started again at the appropriate moment. According to Robba et al. (2016), PEEP of 8 does not cause effects on ICP [20].

Assessments will range five stages: T0 (monitoring start), T1 (before ventilation maneuver for the intervention or control groups), T2 (end of ventilation maneuver our control), T3 (after tracheal aspiration), and T4/T5 (monitoring after 10 and 20 min of procedure). The five initial minutes are required for the initial collection of hemodynamic, ventilation, and neurological data. After the start of this period is T1 with a 10-min duration, followed by T2 and T3, with a 5-min duration each. The individuals in T1 who constituted the MHM group will have their ventilation parameters altered for 10 min according to the lung hyperinflation maneuver adopted, subsequently returning to the baseline values. During this period, the individuals in the control group will be only monitored. In T2, both groups will be subjected to tracheal aspiration; subsequently, data will be monitored for 5 min. Finally, the individuals of both groups were monitored at T4 and T5, 10 and 20 min after the procedure, respectively, to verify vital data stabilization (Fig. 2).

### Experimental group

We will perform ventilation adjustments to proceed with the MHM. The patient will be maintained on dorsal decubitus at the bedside at 30°, on assisted ventilation mode, and controlled volume. Vt will be increased by 150% based on ideal tidal volume; square wave V; inspiratory time (Ti) equal to 1.5 s, adjusted to a peak of inspiratory flow (PIF) lower than the peak of expiratory flow (PEF); RR of 14 irpm, reduced in case of auto-PEEP; PEEP 8 cmH_2_O; FIO_2_ minimum of SpO_2_ of at least 94%. These parameters will be preserved for 10 min. The MV alarms will be adjusted to allow the safety of the maneuver; in addition, in case of Ppeak above 40 cmH_2_O or plateau pressure (Pplateau) above 30 cmH_2_O, the maneuver is immediately interrupted and the previous parameters reestablished. The hyperinflation maneuver will not be associated with any other bronchial hygiene maneuver.

After 10 min, the recommendations of the American Association for Respiratory Care [21] (2010) will be applied in the aspiration procedure, suggesting that the patients are ventilated with FIO_2_ at 100% for 30 s before and 60 s after the procedure to avoid hypoxemia. In this study, each patient will be subjected to tracheal aspiration for 15 s for three consecutive times through a closed aspiration system with catheter number 14. Subsequently, the ventilation parameters will be readjusted according to the initial parameters.

### Control group

The CG will be positioned in dorsal decubitus at 30° and will undergo initial ventilatory adjustments and tracheal aspiration, the same procedures performed in the EG 2 h before the start of data collection. Between T1 and T2, they will only be monitored, not going through the MHM. In T3, they will be aspirated as well as EG. They will undergo non-invasive ICC monitoring at the same moments as the EG.

### Safety assessment and adverse effects

The vital signs (heart rate (HR), RR, MAP, and SpO_2_) will be assessed continuously and registered during the assessment stages through a multiparameter monitor Mindray iMEC12, while capnography will be assessed through a monitor and sensor Dräger Vamos®. Criteria for interrupting the intervention are HR < 60 bpm, systolic blood pressure > 180 mmHg, and diastolic blood pressure < 50 mmHg. The ICP assessed in a non-invasive manner will also be monitored during all procedures and 20 min after the end of the collections. The MV alarms will be adjusted to allow to control the airway pressures.

An expert physiotherapist will be present throughout the collection, and in case of hemodynamic and/or neurological instability, as well as an increase in airway pressures above the allowed values, the collection will be suspended, and a physician of the ICU staff will join the procedure.

### Outcome measures

Primary outcomes will consist of the alterations in ICC indicated in the morphology analysis of the ICP wave due to the MHM since in critical neurological patients, it is crucial to control the secondary lesion and complications caused by the need for MV. Secondary outcomes will range from respiratory mechanics and hemodynamic stability.

### Primary outcome

#### Intracranial compliance

The ICC of all participant patients will be measured in a non-invasive manner through the sensor in the ICP equipment B4C BcMM-R-2000, acquired through funding by the National Council of Research. The researchers were trained by the B4C company and are prepared for data collection. To proceed with the monitoring, a sensor is installed by the researcher coupled to the scalp using an elastic band, without requiring any prior preparation or trichotomy.

The sensor is coupled through wires to a monitor that generates a curve and real-time display of ICP wave, in addition to recording the data for further analysis, which is enabled by extensometers capturing the intracranial volume expansions, since the cranial bone is not entirely hard. Therefore, the pulses are converted into numerical data forming the ICP curve morphology in real time [22].

The ICP wave morphology has its layout, such as in the case of the arterial pulse wave, with a total of up to five distinct peaks, of which three are proper and frequent: percussion wave (P1), tidal wave (P2), and dichroic wave (P3). P1 wave is the most constant in amplitude and derives from the pulse of large cerebral arteries to the choroid plexus. P2 wave, in turn, originates from the cerebral elastance direct reflecting the “reverberation” of P1 on both the brain and cranium, that is, ICC. Finally, the P3 wave is separated from P2 by the dicrotic notch corresponding to the closure of the cardiac aortic valve [23].

The analysis system checks all collected data and analyzes it using a previously created algorithm. The data collected by this sensor are qualitative, which means that the ICP has no absolute value; however, it is possible to analyze the morphology of this pressure curve by extracting three values: P2/P1 ratio, TTP, and pulse amplitude. For this study, all calculations were performed from the average of pulses within each minute of monitoring after excluding possible artifacts. These averages were used to calculate the amplitudes of the two main peaks in the wave morphology, P2 and P1. The P2/P1 ratio was calculated by dividing the amplitudes of the two peaks and the TTP by the time interval from the beginning of each pulse to the peak [15].

In scenarios of normality of ICP and ICC, there is a proportion between the peaks, where P1 > P2. However, in situations of abnormality, there is a change in the morphology of the ICP waveform, with an increase in ICP associated with a low ICC, with P2 > P1. For the TPP variable, the higher the value found, the greater the chance of the peak being P2, which also corresponds to a decrease in the ICC [22, 23].

In this study, the evaluations will be carried out in five (T1–T5) moments:

- T0: start monitoring, the interval between T0 and T1 is intended to ensure patient stabilization for the start of data collection;
- T1: capture initial patient data;
- T2: assess whether the MHM protocol had effects on ICC;
- T3: verify whether aspiration combined with MHM could lead to different effects on outcomes;
- T4 and T5: check whether there was stabilization of parameters related to ICC and respiratory mechanics.

The moment of evaluation T2 is the most relevant for the primary outcome; as for the secondary outcomes, evaluations from T2 to T5 are the most relevant.

Data will be collected through a monitor and analyzed using the Brain4care Analytics System, considering that at a time interval, the software selects an average of pulses to form the waves.

To capture the data, the ICP assessment sensor will be chosen according to the cranial circumference (S, M, L, XL) considering as protocol the lowest sensor possible with reading, following the manufacturer’s recommendations. Data collection will start after the coupling procedure and confirmation by the assessor of reading compatible with the acceptable ICP curves.

### Secondary outcomes

#### Respiratory mechanics

Respiratory mechanics is calculated at all times and for both groups by measuring the variables provided by the MV, which will allow inferring indirectly the efficiency of the maneuvers in promoting bronchial hygiene [7, 8].

The MV parameters will be adjusted according to the predicted weight for each patient [24]. Patients will be ventilated on the volume-controlled assisted mode. The following variables will be recorded: programmed parameters (Vt, PEEP, RR, V, FIO_2_) and variables related to respiratory mechanics [Ppeak, Pplateau, drive pressure, expired current volume, PIF, PEF], which along with the programmed parameters will be applied to calculate resistance (Re), static compliance (StaC), and dynamic compliance (DynC) [25].

StatC, DynC, and Re are clinical variables often used to assess the therapeutic effects of the bronchial hygiene maneuvers [7, 8, 26]. Such effect is possibly related to the recruitment of collapsed lung areas, increase in collateral ventilation, lung areas with a high time constant, and removal of secretions [8].

This protocol will use the following mathematical equations:

1. Predicted body weight
  Male: 50 + 0.91 × (height in centimeters – 152.4)
  Female: 45.5 + 0.91 × (height in centimeters – 152.4)
2. Resistance (Re)
  Re = (Ppeak – Pplateau)/V
3. Static compliance (StaC)
  StaC = VT/(Pplateau – PEEP)
4. Dynamic compliance (DynC)
  DynC = VT/(Ppeak – PEEP)

We will follow a method of flow interruption at the end of the inspiration with a pause of 3 s to calculate the respiratory mechanics. Three measures will be performed using the average of the two measures with the lowest standard deviation. Upon absence of plateau, the measure will be disregarded [8].

#### Hemodynamic stability

The variables of MAP, HR, and SpO_2_ are used in intensive care to assess the hemodynamic impact of respiratory physiotherapy maneuvers [8].

For MAP and HR, we will consider as significant alterations either an increase or decrease by 20 mmHg or 20 bpm, respectively, while for SpO_2_, we will consider values below 90% during the application of the maneuvers [27].

### Sample size

During an extensive literature search, no randomized clinical trials were found that investigated the effect of MHM on CHF in post stroke patients. Thus, it was decided to use a study that investigated the effects of a physiotherapy technique on ICP for the sample calculation.

To obtain the sample size needed for this study, we used the combination of statistical software’s R and G-Power 3.1. First, we extracted the intracranial pressure with the techniques performed in the study bellow [28]. From this previous work, we detected that the median value of ICP for the control group was 16.9, and for the intervention group, it is 11.7. Then, we calculated the Cohen-D effect size from the medians using the package ‘rcompanion’ in R [29]. The final step using the GPower is where we estimate with a power of 95% and a confidence level of 95% the minimum sample size for a *T-Student* equivalence test [30]. The total number of subjects is 60, equally distributed in two groups of 30 patients.

### Statistical methods

The collected data will be organized on a spreadsheet and summarized using techniques of descriptive statistical analysis. The qualitative variables will be summarized by building frequency tables and the quantitative variables by calculating the descriptive measures (average, mean, standard deviation, and percentiles 25–75).

The evaluations’ primary outcome and secondary outcomes will be made in 5 different times; for that, the data will be analyzed by analysis of variance (ANOVA) two-way for repeated measures with the Tukey post-test for results that present normal distribution and homogeneity of variances, verified by the test of Shapiro–Wilk and Average in Levene, respectively.

We will be using the statistical software Statistical Package for the Social Sciences (SPSS) (IBM—version 22) to analyze the collected data. Our research hypotheses will be tested using non-parametric methods. Wilcoxon test with Kruskal–Wallis post-test will also be applied (intergroups). The level of significance established was *p* ≤ 0.05. The evaluation of results and data analysis will be carried out by an independent professional.

### Methods in analysis to handle protocol non-adherence and any statistical methods to handle missing data

Data will be analyzed on an intention-to-treat basis to include all participants based on the random allocation regardless of whether the intervention has been received or not. Adherence to intervention or withdrawal, which is recorded by our trained research assistants on a weekly basis, serves as a covariate and will be included in the data analysis.

Missing data will not be imputed for main primary and secondary analyses. As sensitivity analyses, these analyses will be repeated, using multiple imputation with chained equations for missing data.

### Composition of the data monitoring committee, its role and reporting structure

The principal investigator and co-investigators will monitor the data collection and storage to ensure that the data is kept and used in accordance with the protocol. A statistician, who is independent and without competing interests, will be responsible to inspect clinical data collected during the study period.

### Frequency and plans for auditing trial conduct

Led by the study coordinator, regular trial conduct, procedural checks, and quality assurance checks will be completed by the study staff. They will verify that all documents containing protected health information are secured and align with the procedures outlined in the protocol.

### Dissemination plans

We aim to publish results from the study in international peer-reviewed journals brain injury rehabilitation. The full protocol, participant-level dataset, and statistical code are granted public access. Authorship will be determined in line with the International Committee of Medical Journal Editors guidelines.

### Ethical considerations

All data will only be accessed by the study researchers and kept strictly confidential. Patient data will be protected by locked cabinets, and use of passwords limited access storage of electronic data. Data will be collected on paper forms and scanned in sequence. As a way to avoid errors, they will be double-checked when they are scanned.

## Discussion

Stroke is among the acute cerebral lesions that present the highest morbidity and mortality in the world and has been increasingly growing [2]. These patients are admitted to intensive care units due to the severity of the lesion and need for MV, which is related to the lesion region and consequent deficits in airway protection, level of awareness, and respiratory control [2].

Patients under MV have a high risk of bronchial secretions retention. Furthermore, tracheal intubation is related to lower effectiveness and cough reflex, interruption of mucociliary system, and rheologic modification of the mucus, in addition to immobility imposed on the patient, general weakness, and fluid restriction, which can contribute to enhancing the viscosity of the mucus and need for bronchial hygiene maneuver [1, 31–34].

When applied on the chest, these maneuvers increase the ITP, with a decrease in venous return, heart debt, and MAP [35]. Therefore, techniques applied in physiotherapy can cause transitory hemodynamic alterations, including in the ICP [1, 36]. The difference between MAP and ICP characterizes cerebral perfusion pressure, and the pressure gradient enables blood circulation in the intracranial constituents [1]. Thus, alterations in any of these components without increasing or reducing the others can worsen the cerebral lesion by hypoxic-ischemic involvement [37]. In this way, the relationship between the cardiorespiratory and cerebral systems is evident [1].

The analysis of the ICP waveform and the respiratory variations during the tracheal aspiration procedure showed that during the procedure, there is a relevant brain response to changes in cranial volume, which can be explained by an increase in intrathoracic pressure [35].

Recent studies demonstrate the importance of neurological monitoring in the outcome of neurocritical patients [38]. The analysis of the average of the ICP pulses, by the morphology of the waveform, makes it possible to obtain the data of the P2/P1 ratio and the TTP, which characterizes the ICC. ICC seems to better reflect intracranial homeostasis than the absolute value of ICP itself [39].

This is the first randomized clinical protocol to propose and verify the effectiveness of an MHM and its influence on the ICC of critical neurological patients through a non-invasive assessment of ICC, supported by an extensive literature review.

With this study, we expect to demonstrate the safety of performing hyperinflation maneuvers under a mechanical ventilator in critical neurological patients, proving to be a respiratory physiotherapy resource that can be used in clinical practice and provide benefits to respiratory mechanics without jeopardizing the neurological function.

## Trial status

At the time of manuscript submission, the study is in the recruitment phase. Recruiting started in March 26, 2019, and will continue until the target sample size is reached, which is expected to occur during November 2022.

## Supplementary Information

**Additional file 1.** SPIRIT Checklist for *Trials.*

## Data Availability

The datasets used and/or analyzed during the current study are available from the corresponding author on reasonable request.

## Abbreviations

B4C: Brain4care
CG: Control group
CO: Cardiac output
CO_2_: Carbon gas
DD: Dorsal decubitus
DynC: Dynamic compliance
EG: Experimental group
ETCO_2_: End-tidal CO_2_
FIO_2_: Inspired oxygen fraction
HR: Heart rate
IC: Informed consent
ICC: Intracranial compliance
ICP: Intracranial pressure
ICU: Intensive care units
ITP: Intrathoracic pressure
MAP: Mean arterial pressure
MHM: Mechanical hyperinflation maneuver
MV: Mechanical ventilation
PEEP: Positive expiratory-end pressure
PEF: Peak of expiratory flow
PIF: Peak of inspiratory flow
Ppeak: Peak pressure
Pplateau: Plateau pressure
RASS: Richmond Agitation-Sedation Scale
Re: Resistance
RR: Respiratory rate
SpO_2_: Peripheral oxygen saturation
StaC: Static compliance
Ti: Inspiratory time
am: Time to peak
V: Flow
Vt: Tidal volume
